# Visual Dependency and Dizziness after Vestibular Neuritis

**DOI:** 10.1371/journal.pone.0105426

**Published:** 2014-09-18

**Authors:** Sian Cousins, Nicholas J. Cutfield, Diego Kaski, Antonella Palla, Barry M. Seemungal, John F. Golding, Jeffrey P. Staab, Adolfo M. Bronstein

**Affiliations:** 1 Neuro-otology Unit, Division of Brain Sciences, Imperial College London, Charing Cross Hospital, London, United Kingdom; 2 Interdisciplinary Centre for Vertigo & Balance Disorders, Department of Neurology, Zürich, Switzerland; 3 Department of Psychology, University of Westminster, London, United Kingdom; 4 Department of Psychiatry and Psychology, Mayo Clinic, Rochester, Minnesota, United States of America; University of British Columbia, Canada

## Abstract

Symptomatic recovery after acute vestibular neuritis (VN) is variable, with around 50% of patients reporting long term vestibular symptoms; hence, it is essential to identify factors related to poor clinical outcome. Here we investigated whether excessive reliance on visual input for spatial orientation (visual dependence) was associated with long term vestibular symptoms following acute VN. Twenty-eight patients with VN and 25 normal control subjects were included. Patients were enrolled at least 6 months after acute illness. Recovery status was not a criterion for study entry, allowing recruitment of patients with a full range of persistent symptoms. We measured visual dependence with a laptop-based Rod-and-Disk Test and severity of symptoms with the Dizziness Handicap Inventory (DHI). The third of patients showing the worst clinical outcomes (mean DHI score 36–80) had significantly greater visual dependence than normal subjects (6.35° error vs. 3.39° respectively, p = 0.03). Asymptomatic patients and those with minor residual symptoms did not differ from controls. Visual dependence was associated with high levels of persistent vestibular symptoms after acute VN. Over-reliance on visual information for spatial orientation is one characteristic of poorly recovered vestibular neuritis patients. The finding may be clinically useful given that visual dependence may be modified through rehabilitation desensitization techniques.

## Introduction

Vestibular neuritis (VN) is an acute disorder characterised by vertigo, nausea, vomiting and imbalance. VN is a leading cause of acute vertigo in general practice and emergency departments. [1] Although the aetiology is not firmly established, VN is thought to arise through viral-mediated inflammation of the vestibular nerve. [2] Clinical recovery is partly related to regaining vestibular nerve activity peripherally and partly mediated by a central process of vestibular compensation. [3] Typically, symptoms last for a few days or weeks but for reasons that are not fully clear, up to a half of patients continue to suffer from symptoms of dizziness, unsteadiness and spatial disorientation long after recovery from their acute illnesses is expected. [4], [5] Although these chronic symptoms are not severe or life threatening, they generate significant personal and social handicap in patients [6], [7], leading to frequent consultations in general practice, ENT and neurology clinics. [8] Thus, identifying the possible mechanisms responsible for insufficient clinical recovery following acute VN should be a research priority.

Several pathological processes have been postulated to be responsible for long term vestibular symptoms following acute VN, including the effects of persistent peripheral vestibular deficits, incomplete central vestibular compensation, psychological morbidity, and continued over-reliance on visual signals. The degree of recovery of peripheral vestibular function has much less effect than was thought in decades past, with the majority of the literature showing poor correlations between reflex indicators of peripheral vestibular function and chronic symptoms. [5], [9]–[13] In agreement, treatment of acute VN with corticosteroids, which enhances recovery of peripheral vestibular function [14], [15], does not improve long term symptomatic recovery. [15]–[17] However, one report found that a positive head impulse test was associated with worse clinical outcome [18] indicating that patients with particularly large acute vestibular deficits may have more difficulty with recuperation.

Vestibular compensation is a broad term encompassing various centrally mediated processes from brainstem to cortex that restore vestibular reflex symmetry and postural performance, and adaptively modulate responsiveness to visual and proprioceptive stimuli. [19]–[21] Animal models have elucidated a stepwise process of compensation at the level of the vestibular nuclei and cerebellar pathways. [22], [23] In humans, structural and functional MRI studies have correlated cortical changes with clinical disability in patients with VN and acoustic neuromas. [24]–[26] Vestibular compensation also involves perceptual processes [27], which correlate better with symptom outcomes than the severity of peripheral deficits. [28] However, there is no convincing evidence explicitly linking differences in central compensation to the extent of recovery from clinical symptoms.

Retrospective and prospective studies suggest that pre-existing anxiety disorders [29], [30], anxious/introverted personality traits [31], and poor self-efficacy [32] may be risk factors for persistent vestibular symptoms following acute vestibular events. Also, high anxiety [33], [34] and catastrophic interpretations of bodily sensations [34] during acute bouts of VN predict poor long term outcomes. Indeed, these psychological factors are the foundations of the clinical syndromes of phobic postural vertigo [35] and chronic subjective dizziness [36] that have been used to describe patients with persistent dizziness following acute vestibular events. However, the absence of biomarkers that might explicate relationships among the physical and psychological components of these syndromes has hampered efforts to validate them.

Normal spatial orientation and locomotor control require proper integration of vestibular, visual and somatosensory stimuli, but there is a natural variability in the relative weightings that individuals accord to these three sources of information. The degree of reliance on visual stimuli, known as visual or field dependence is distributed normally in the general population. Witkin and Asch first demonstrated this in 1948 using the Rod and Frame Test. [37] They showed that normal individuals (not patients) who were more visually dependent made greater errors in aligning a rod with the earth vertical axis when it was enclosed within a tilted frame. Later investigators measured visual dependence using the Rod and Disk Test, in which a rotating disk replaced the tilted frame in the visual background [38]–[40] or posturography with a moving visual surround [41].

There is no evidence that individuals with naturally high levels of visual dependence have difficulty with locomotion under normal circumstances. However, elevated visual dependence was identified in patients with chronic visual vertigo that followed a variety of acute peripheral and central vestibular illnesses. [39], [42], [43] The relationship between visual dependency and clinical handicap in VN patients, *unselected* for the presence of visually induced symptoms, is unknown. Indeed, previous studies showing increased visual dependency in mixed chronic vestibular patients have not been outcome-related [41].

The present study is therefore the first to investigate a possible link between visual dependence and long term symptomatic outcomes specifically in patients with well established VN. We recruited patients with clinical and laboratory evidence of VN occurring 6 months or more prior to study entry. The study included subjects with a full range of outcomes from complete recovery to severe chronic symptoms, providing an unbiased opportunity to investigate visual dependence across a full range of long term clinical outcomes. We measured visual dependence using the Rod and Disk Test with stationary and moving backgrounds and symptom severity with the Dizziness Handicap Inventory (DHI). We hypothesised that patients with poorer clinical outcomes would have higher levels of visual dependence than those with better outcomes and normal control subjects. Such a finding would have clinical and research implications. Clinically, it would provide a means of individualizing treatment by identifying patients most likely to benefit from visual desensitization exercises. Scientifically, it would suggest an avenue for further investigations of a potential mechanism underlying poor recovery.

## Subjects and Method

Twenty-eight patients (mean age 51, range 22–75, Females 10) with clinical histories, physical examinations and laboratory testing typical of acute VN were recruited. All 28 patients recruited were tested in the chronic stage of VN (a minimum of 6 months after acute VN onset).Twenty-four of these patients were seen acutely by the authors, but were not tested until they had reached the chronic phase (>6 months after acute onset). Acutely, these patients presented with new onset vertigo and were found to have spontaneous horizontal nystagmus with a torsional component, unilaterally positive head impulse tests, and otherwise normal neurological examinations. Unilateral canal paresis was confirmed by deficits of at least 20% on bithermal caloric testing (range 21–100%).[44] Audiometric assessment ruled out cochlear involvement. The remaining 4 patients were recruited and tested after attending an outpatient Neuro-otology clinic (acute VN onset >6 months previous). Acute assessment by the authors for these additional 4 patients was not available and so these patients were diagnosed on the basis of typical histories of VN, medical record documentation of acute findings and examinations, including normal audiograms, and bithermal caloric testing confirming canal paresis >20% (range 35–75%) and lack of any additional neurological or oculo-motor abnormality. Symptom severity was not a study selection criterion (i.e., potential subjects were screened and enrolled on the basis of their VN history, not their symptoms). Twenty-five individuals (mean age 45 years, range 29–72, females 11) with no histories of neuro-otological problems or visual deficits apart from corrected refractive errors were enrolled as normal control subjects. Control subjects did not undergo caloric testing. There were no significant differences in mean age or gender distribution between patient and control groups.

**Figure 1:**
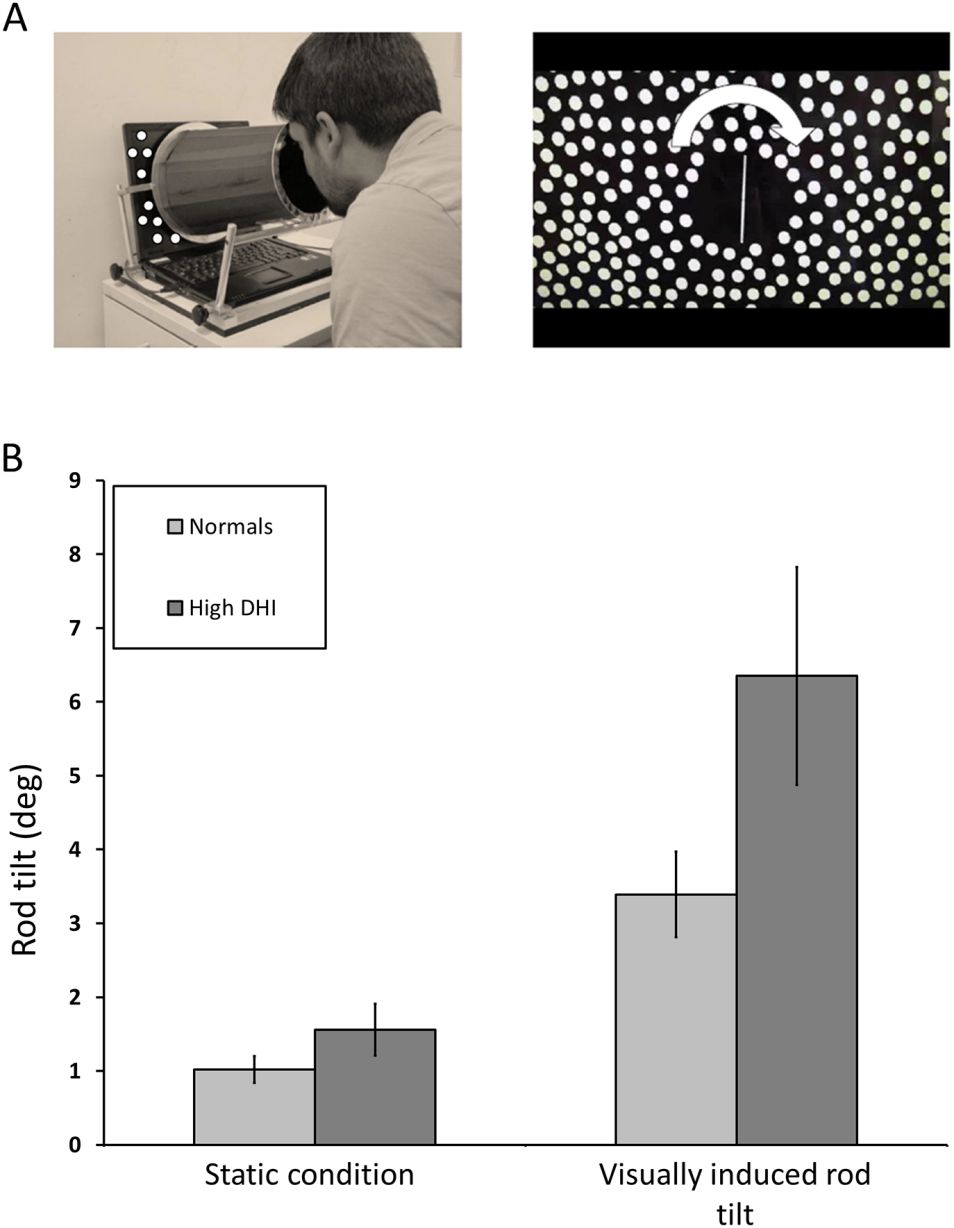
Experimental set up and rod tilt in normals and high DHI patient group. A. Rod and Disk test experimental set up. Laptop-based Rod-and-Disk test to measure visual dependency, showing a subject viewing the screen through a field-restricting cone. Subjects carried out the test in a darkened room. **B.** Rod tilt in normals and high DHI patient group. Figure showing similar mean rod tilt (deg; ± SE) in the static condition for the normal control and high DHI patient groups. Also shown is visually induced rod tilt for both normal and High DHI groups, which is higher in the unrecovered patient group, despite similar values in the static condition.

Symptom severity was measured with the Dizziness Handicap Inventory (DHI), a validated 25-item questionnaire that assessed physical and emotional symptoms and functional impairment due to dizziness. [45] Visual dependence was measured with the Rod and Disk Test [38] on a laptop computer (Figure 1A). Subjects were seated in front of the computer in a darkened room with their heads held against a viewing cone that blocked extraneous visual orientation cues. The diameter of the cone at the subjects’ eyes was 15 cm with a depth of field of 30 cm, subtending a viewing angle of 39°. The visual stimulus consisted of a luminous white 6 cm rod on a black background. The rod rotated 360° in either direction about its midpoint in the central 11° of the visual filed. Outside of this central zone, the viewing screen was filled with a collage of 220 off-white dots, each 8 mm (1.5° of visual field) in diameter, randomly distributed on a black background. Subjects controlled the orientation of the rod with a roller mouse. They were instructed to align the rod to their perceived vertical (the subjective visual vertical) under three conditions. In condition 1, the collage of dots was stationary. In conditions 2 and 3, the collage rotated clockwise or counterclockwise, respectively, at 30°/s. Subjects were given four trials in each condition, with conditions 2 and 3 presented in random order after condition 1. During each trial the rod was initially set randomly at ±40° from vertical. The rod tilt for each trial was recorded as the difference in degrees between true vertical and the subjects’ final placement of the rod. (Rod-and-Disk software is available online at: http://www.imperial.ac.uk/medicine/dizzinessandvertigo).

For statistical analysis, the patients were divided into three subgroups based on symptom severity. Subgroups were identified by visual inspection of the distribution of DHI scores and corresponded to clinically meaningful subsets (Figure S1, A): (i) Asymptomatic, fully recovered (DHI = 0; n = 9), (ii) Low symptoms (DHI 2–28; n = 10), (iii) High symptoms (DHI 36–80; n = 9). Visually induced rod tilt was calculated as a measure of visual dependence for each subject. First, static tilt was calculated as the mean rod tilt in the four trials of condition 1 (disk static). Then, visually induced rod tilt was calculated as the mean of the absolute values of the rod tilt from each trial of conditions 2 and 3 (disk moving) minus the static rod tilt.

Visually induced and static rod tilts were compared between patient and control groups, and across patient subgroups with one-way ANOVAs, followed by pairwise comparisons of subgroups. Secondary analyses tested associations between severity of caloric paresis, DHI scores and visually induced rod tilts to identify possible contributors to group differences. Visually induced rod tilt was also calculated separately for contralesional and ipsilesional rotations. Visually induced rod tilts and DHI scores were not normally distributed, but repeating the statistical tests after logarithmic transformations and using Wilcoxon tests for pairwise comparisons yielded the same results as the original parametric analyses. For information, non-parametric results are shown alongside ANOVA results. Statistics were carried out in SPSS (IBM SPSS Statistics version 21) and significance level used was P<0.05.

### Ethics Statement

The Charing Cross Research Ethics Committee, London, approved this study (MRC award Program Number G0600183). Written informed consent was obtained from all subjects.

## Results

**Figure 2:**
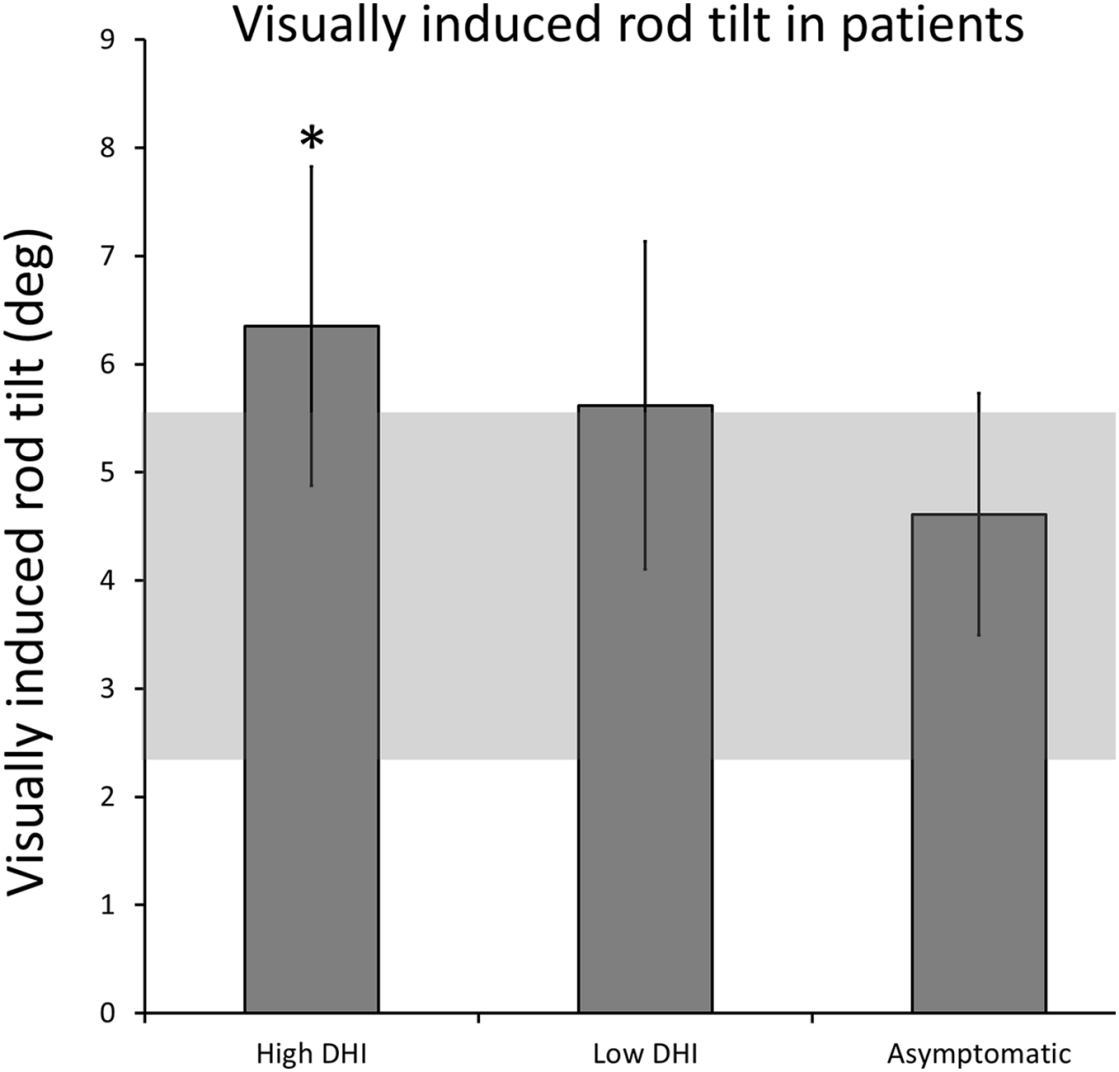
Visually induced rod tilt for all patient groups and normals. Figure showing visually induced rod tilt (mean, ±SE) for all patient groups (High DHI; Low DHI; Asymptomatic). Shaded grey area represents 95% confidence interval of the mean for normal controls. Note, rod tilt values for Low DHI and Asymptomatic patient groups are within normal range, where as High DHI patients show significantly higher than normal rod tilts in the moving disk condition.

Visually induced rod tilts were always in the direction of disk rotation, as expected, for all subject groups. Figure 1B shows rod tilts in the control group and high DHI patient subgroup. Note that static rod tilts were small and similar in these two subject groups whereas visually induced rod tilts were nearly double in the high DHI group. Figure 2 depicts mean visually induced rod tilt for the three patient subgroups. Only the most symptomatic subgroup had a mean visually induced rod tilt that exceeded the 95% confidence interval of the control subjects.

Group mean static rod tilts were not statistically different between the control group and any of the three patient subgroups. In contrast, group mean visually induced rod tilts were larger and differed significantly between the patient group as a whole (5.51SD4.14°) and the control group (3.39SD2.9°) [F(1,51) = 4.6, p = 0.04; z = –2.32, p = 0.02]. Pairwise comparisons of patient subgroups with the control group identified significantly greater visually induced rod tilt in the High DHI subgroup (6.35°SD4.43) compared to controls (3.39°SD2.92) [F(1,32) = 5.14, p = 0.03; z = 2.05, p = 0.04]. Despite this, within the patient group as a whole, there was no significant correlation between visually induced rod tilts and DHI scores.

Secondary analyses found a marginal correlation between severity of canal paresis and DHI scores (Figure S1, B: Pearson’s r = 0.36, p = 0.06; Spearman’s r = 4.1, p = 0.03), but no differences in mean canal paresis among patient subgroups [F(2,24) = 2.26, p = 0.13; *x^2^*(2) = 3.9, p = 0.14]. Furthermore, there was no relationship between severity of canal paresis and visually induced rod tilt (Figure S1, C). Visually induced rod tilt was similar for both contralesional (6.26°SD4.57) and ipsilesional (6.31°SD4.74) disk rotations.

## Discussion

In this study, increased visual dependence, as measured by visually induced rod tilt on the Rod and Disk Test, was associated with the severity of persistent (>6 months) vestibular symptoms in patients who had experienced acute VN. The association between visual dependence and severity of symptoms was not linear, but found in the subgroup of patients with the poorest recovery. These patients rated their symptoms at a moderate to severe level that was associated with functional impairment in daily living. In contrast, their asymptomatic counterparts and those with only low level symptoms did not demonstrate elevated visual dependence.

It is possible that patients with naturally high premorbid levels of visual dependence are inherently susceptible to developing persistent vestibular symptoms after acute VN. This hypothesis cannot be tested directly. Alternatively, patients with persistent symptoms may be ones who develop excessive visual dependence as a consequence of their acute vestibular illnesses. These possibilities cannot be distinguished in a cross-sectional investigation such as this one, but require a prospective study, particularly one with evaluations before and after successful treatment.

The secondary analyses shed some light on potential mechanisms. As in previous studies, there was little evidence of a relationship between canal paresis and persistent symptoms. [13] Furthermore, there was no association between canal paresis and visual dependence. In agreement with Kim et al [18], static subjective visual vertical results in our VN patients was not predictive of long term clinical outcome indicating that spatial perception was adequate in static environments. There is no doubt that acute VN was the event that precipitated our patients’ symptoms, but their residual peripheral deficits cannot be the sole perpetuating factor. This leaves open the possibility that compensatory mechanisms had not properly restored higher order perceptual functions, leaving patients vulnerable to subjective sensations of dizziness, unsteadiness, and vulnerability in challenging motion environments.

Psychological factors were not examined in this study. Previous research has indicated that pre-existing anxiety diatheses and high levels of acute anxiety at the time of acute vestibular events may presage persistent vestibular symptoms [29]–[31], [33], [34], but the role of psychological factors over the long term is not as clear, apart from non-specific associations between psychological distress and chronic symptoms that have been observed in all major medical illnesses. Even in patients diagnosed with the behaviourally mediated syndromes of phobic postural vertigo and chronic subjective dizziness, psychiatric morbidity is not universal as 25% of individuals with these conditions do not have diagnosable psychiatric disorders or chronically elevated levels of anxiety or depression. [46], [47] Therefore, psychological factors may predispose and precipitate chronic symptoms after acute VN, but their potential roles as specific perpetuating mechanisms are much less clear.

In the setting of acute VN, one would expect patients to increase the weighting given to visual cues as an adaptive mechanism to counter inaccurate vestibular information. [48] However, the intrinsically ambiguous nature of visual information, signalling motion of both self and surroundings, would dictate that patients revert back to a non-visually dependent (i.e. inertially based) mode of spatial orientation and locomotor control as quickly as possible to promote full recovery. Indeed, animal models show that the increased use of visual cues after unilateral vestibular injury declines normally over the compensation period due to central nervous system plasticity. [49] Our findings showing increased visual dependence in highly symptomatic patients long after the typical central compensation period suggest that failure to revert to a more natural three-way integration of vestibular, visual and somatosensory information may be a perpetuating mechanism underlying poor outcomes.

It is unlikely that any single mechanism promotes full recovery or perpetuates ongoing symptoms after acute vestibular events such as VN. Interactive processes involving perceptual and psychological components are more plausible. As an example, it has been hypothesized that activation of anxiety systems by acute vertiginous states promotes prolonged overreliance on visual or somatosensory cues in vulnerable individuals [50], a concept consonant with the present results. Testing of hypotheses like this will require prospective studies that reliably measure multiple factors, including psychological measures that may contribute to poor outcomes, in a manner that will permit analyses of their interactions over time. From a clinical standpoint a simple laptop-based Rod and Disk Test may act as a biomarker for high levels of dizziness mediated handicap in patients following bouts of acute VN. This is important as visual motion desensitization techniques are available to treat refractory dizziness in chronic vestibular patients [51].

## Supporting Information

Figure S1 **A.** Scatter plot showing visual dependency and symptom load (DHI). Figure showing visually induced rod tilt (degrees) and DHI score in patients. Grey dashed lines show patient sub groups as defined by DHI score. **B.** Scatter plot showing canal paresis and symptom load (DHI). Figure showing vestibular canal paresis (%) and DHI score in patients. **C.** Scatter plot showing canal paresis (%) and visual dependency. Figure showing vestibular canal paresis (%) and visually induced rod tilt in patients.
(TIF)
